# Overexpression of the Tibetan Plateau annual wild barley (*Hordeum spontaneum*) *HsCIPKs* enhances rice tolerance to heavy metal toxicities and other abiotic stresses

**DOI:** 10.1186/s12284-018-0242-1

**Published:** 2018-09-12

**Authors:** Weihuai Pan, Jinqiu Shen, Zhongzhong Zheng, Xu Yan, Jianxin Shou, Wenxiang Wang, Lixi Jiang, Jianwei Pan

**Affiliations:** 1 MOE Key Laboratory of Cell Activities and Stress Adaptations, School of Life Sciences, Lanzhou University, Lanzhou, 730000 China; 2 College of Life Sciences, Shaoxing University, Shaoxing, 312000 Zhejiang China; 3 College of Chemistry and Life Sciences, Zhejiang Normal University, Jinhua, 321004 Zhejiang China; 4 Department of Agronomy, College of Agriculture and Biotechnology, Zhejiang University, Hangzhou, 310058 China

**Keywords:** Abiotic stresses, Cloning, Heavy metal toxicity, *HsCIPKs*, Overexpression, Tibetan plateau annual wild barley, Rice, Transformation

## Abstract

**Background:**

The calcineurin B-like protein (CBL) and CBL-interacting protein kinase (CIPK) signaling system plays a key regulatory role in plant stress signaling. The roles of plant-specific CIPKs, essential for CBL-CIPK functions, in the response to various abiotic stresses have been extensively studied so far. However, until now, the possible roles of the *CIPKs* in the plant response to heavy metal toxicities are largely unknown.

**Results:**

In this study, we used bioinformatic and molecular strategies to isolate 12 *HsCIPK* genes in Tibetan Plateau annual wild barley (*Hordeum spontaneum* C. Koch) and subsequently identified their functional roles in the response to heavy metal toxicities. The results showed that multiple *HsCIPKs* were transcriptionally regulated by heavy metal toxicities (e.g., Hg, Cd, Cr, Pb, and Cu) and other abiotic stresses (e.g., salt, drought, aluminum, low and high temperature, and abscisic acid). Furthermore, the ectopic overexpression of each *HsCIPK* in rice (*Oryza sativa* L. cv Nipponbare) showed that transgenic plants of multiple *HsCIPKs* displayed enhanced tolerance of root growth to heavy metal toxicities (Hg, Cd, Cr, and Cu), salt and drought stresses. These results suggest that *HsCIPKs* are involved in the response to heavy metal toxicities and other abiotic stresses.

**Conclusions:**

Tibetan Plateau annual wild barley *HsCIPKs* possess broad applications in genetically engineering of rice with tolerance to heavy metal toxicities and other abiotic stresses.

**Electronic supplementary material:**

The online version of this article (10.1186/s12284-018-0242-1) contains supplementary material, which is available to authorized users.

## Background

In plant cells, the calcium ion (Ca^2+^) is involved as a second messenger in the regulation of a variety of abiotic and biotic stress responses and the formation and development of plant organs (Dodd et al. 2010). The core components of Ca^2+^ signaling are calcium sensors, including calmodulins (CaMs), calmodulin-like proteins (CMLs), calcium-dependent protein kinases (CDPKs), and calcineurin B-like proteins (CBLs), which bind Ca^2+^ and activate downstream signaling components (Rudd and Franklin-Tong 1999; Singh and Parniske 2012; Zheng et al. 2013). Among these Ca^2+^-bound calcium sensors, CBLs selectively interact with plant-specific CBL-interacting protein kinases (CIPKs) and thereby form a CBL-CIPK signaling system that has been demonstrated to serve as a key regulation node during stress signaling in plants (Luan 2009; Weinl and Kudla 2009; Shen et al. 2014). Thus, dissecting the mechanisms of the CBL-CIPK signaling system is one of the research priorities in the plant stress physiology field. Due to the lack of kinase activity in CBLs, different combinations with CIPKs largely determine the specificity, diversity, and complexity of the CBL-CIPK signaling system (Batistic et al. 2010**)**. Therefore, functional identification of the CIPKs in distinct plant species will enhance the better understanding of the functional roles and modes of action of the CBL-CIPK signaling system.

Bioinformatic analysis has shown that there are 26 and 31 *CIPK* homologous genes in the model plant genomes of *Arabidopsis thaliana* and rice (*Oryza sativa*), respectively (Kolukisaoglu et al. 2004). Recently, multiple *CIPK* families have been bioinformatically identified in other plant species, including poplar (*Populus*) (Yu et al. 2007), cotton (*Gossypium spp*) (Wang et al. 2016a), soybean (*Glycine max*) (Zhu et al. 2016), canola (*Brassica napus*) (Zhang et al. 2014), eggplant (*Solanum melongena*) (Li et al. 2016), cassava (*Manihot esculenta*) (Hu et al. 2015), maize (*Zea mays*) (Chen et al. 2011), and wheat (*Triticum aestivum*) (Sun et al. 2015). However, genomic analysis of the cultivated barley (*Hordeum vulgare*) *HvCIPK* family remains lacking. Tibetan plateau wild barley, in particular, the annual wild barley (*Hordeum spontaneum* C. Koch), has suffered the extreme climate and environmental conditions for a long term and therefore has evolutionally generated abundant natural variations and/or unique gene networks for stress tolerance. Due to the close genetic homology of Tibetan plateau wild barley to cultivated barley, the Tibetan plateau was recently considered to be one of the centers of domestication of cultivated barley (Dai et al. 2012). Thus, Tibetan plateau wild barley is one of the few germplasm resources to utilize wild barley *CIPKs* to genetically engineer rice or other crops with higher stress tolerance.

Although CIPKs have been demonstrated to function in various responses to abiotic stresses, including salt, drought, flood, wounding, abscisic acid (ABA), low and high temperature (Guo et al. 2001; Kim et al. 2003; Lee et al. 2009; Li et al. 2012; Yan et al. 2014; Zhang et al. 2014), biotic stresses, such as pathogen infection (Kurusu et al. 2010; de la Torre et al. 2013; Meteignier et al. 2017), and nutrient deficiency (Xu et al. 2006; Pandey et al. 2007; Wang et al. 2016b; Straub et al. 2017), so far, no evidence has shown that CIPKs are involved in the plant response to heavy metal toxicities, which is one of the most dangerous types of toxic species for plants and therefore for animals and humans via the food chain. Heavy metals are defined as elements having a specific gravity above five and include mercury (Hg), cadmium (Cd), chromium (Cr), copper (Cu), and lead (Pb). Heavy metals-polluted soils cause irreversible harm to plant growth and development, and crop yield and quality (Mustafa and Komatsu 2016) due to their extremely stable and nonbiodegradable biochemical characteristics. Heavy metal ions enter the cell and tightly bind to intracellular protein enzymes by replacing specific cations from their binding sites, leading to the inactivation of enzymes and the induction of reactive oxygen species (ROS), (Sharma and Dietz 2009), which causes oxidative damage to plant cells. Recent studies have shown that Ca^2+^ or Ca^2+^-dependent signaling is involved in plant tolerance to heavy metal stresses, including Cd and Cr (Fang et al. 2014; Huang et al. 2014; Ahmad et al. 2015), and aluminum (Al) toxicity (Zhang and Rengel 1999; Lan et al. 2016). However, whether CIPKs function in Ca^2+^-dependent plant tolerance to heavy metal and Al toxicities is largely unknown.

In this study, we functionally identified the roles of 12 members of the Tibetan plateau annual wild barley *HsCIPK* family in the response to heavy metal toxicities, including Hg, Cd, Cr, Cu, and Pb, and other abiotic stresses such as salt, drought, Al, low and high temperature, and ABA. Our results demonstrate that multiple *HsCIPKs* are involved in plant tolerance to multiple heavy metal toxicities and salt and drought stresses.

## Methods

### Plant materials and growth conditions

Grains of wild barley, Tibetan Plateau annual wild barley X74 (*Hordeum spontaneum* C. Koch), and Nipponbare rice (*Oryza sativa* L. ssp. *japonica*) were surface sterilized by 70% ethanol for 10 min followed by 10% NaClO for 30 min and finally 8 rinses with water. The endosperms in barley and rice grains contain a large amount of nutrients and therefore provide enough nutrients to sustain the grains for one week following germination. Thus, a simple CaCl_2_ solution (0.1 mM CaCl_2_; pH 5.8; Pan et al. 2004) was used for barley and rice grain germination and seedling growth. To germinate the barley grains (Pan et al. 2004), sterilized grains were germinated between two layers of wet filter papers with the CaCl_2_ solution for one day under darkness (25 °C), and the germinated grains were incubated for another four days in the CaCl_2_ solution under darkness (25 °C). To germinate the rice grain (Pan et al. 2011), sterilized grains were treated for three days under darkness (4 °C) and subsequently incubated for 3 days at 37 °C for germination. Finally, the germinated rice grains were transferred to the CaCl_2_ solution and incubated for four days under light conditions (14-h light/10-h dark, 28 °C light/25 °C dark). Four-day-old barley and rice seedlings with similar root lengths were used in this study.

### In silico cloning, molecular cloning, and sequence analysis of *HsCIPKs*

Two approaches were used in *HsCIPK* in silico cloning. We first used available full-length cDNAs of rice *OsCIPK1* to *OsCIPK31* (http://www.ncbi.nlm.nih.gov/) (accession numbers of the *OsCIPK* cDNAs are shown in Additional file 1: Table S1) as probes to search for their homologous genes in a full-length cDNA library of a barley cultivar (http://earth.lab.nig.ac.jp/~dclust/cgi-bin/barley_pub/). Second, full-length cDNAs, conserved motif in kinase domain, and the NAF/FISL domain motif of rice *OsCIPK1* to *OsCIPK31* as probes were subjected to a BLAST comparison with the barley nucleotide collection (nr/nt) database (http://blast.ncbi.nlm.nih.gov/) for their homologous fragment sequences, and the subsequent resulting homologous fragment sequences were spliced in silico and extended for multiple rounds via corresponding overlapping contigs. Finally, the resulting full-length cDNAs were analyzed by DNA STAR SeqMan and Megalign software.

Reverse transcription-polymerase chain reaction- (RT-PCR-) and sequencing-based approaches were used to clone the *HsCIPK* coding sequences (CDSs) from a total cDNA pool of the Tibetan Plateau annual wild barley X74. *HsCIPK*-specific primers for the RT-PCR assay (Additional file 1: Table S2) were designed based on the recovered in silico cDNA sequences of the *HvCIPKs*. Furthermore, 5’-RACE (rapid amplification of cDNA ends) and 3’-RACE were used to confirm individual 5′- and 3′-end sequences. Finally, the resulting full-length cDNAs of individual *HsCIPKs* were analyzed by sequence alignment and open reading frame (ORF) comparison with *OsCIPKs* and *AtCIPKs* (Accession numbers of *AtCIPK* cDNAs shown in Additional file 1: Table S1). Using the Clustal W Method in DNA STAR Megalign software, cDNA sequence-based predicted amino acid sequences were used to generate a phylogenetic tree of HsCIPKs, OsCIPKs, and AtCIPKs.

### Chemicals and treatments

Unless specified, all reagents were from Sigma-Aldrich. All chemical stock solutions were prepared as follows: water was used to dissolve CaCl_2_ (1 M), NaCl (5 M), AlCl_3_ (40 mM), HgCl_2_ (0.5 M), CdCl_2_ (10 mM), PbCl_2_ (10 mM), K_2_Cr_2_O_7_ (0.5 M), and CuSO_4_ (1 M). Abscisic acid (ABA; 10 mM) was first dissolved in a few drops of 1 M KOH and then diluted with water, whereas polyethylene glycol 6000 (PEG 6000) powders were directly prepared before use (see below). Unless otherwise indicated, in the qRT-PCR analysis of the transcriptionally induced expression levels of the endogenous *HsCIPKs* under heavy metal toxicities and other abiotic stresses, the final working concentrations were 400 mM for NaCl, 1 mM for CuSO_4_, 0.5 mM for K_2_Cr_2_O_7_, 20 μM for HgCl_2_, PbCl_2_, CdCl_2_ AlCl_3_, and ABA, and 20% (*w*/*v*) for PEG 6000. In the root growth assay of rice transgenic lines, based on the effects of different concentrations on root elongation in the wild-type Nipponbare rice (Additional file 1: Figure S1), the final working concentrations were 50 mM for NaCl, 50 μM for AlCl_3_, 5 μM for K_2_Cr_2_O_7_, PbCl_2_, and CdCl_2_, 1 μM for ABA, 0.5 μM for HgCl_2_, 0.25 μM for CuSO4, and 10% (*w*/*v*) for PEG 6000. All stock solutions of these chemicals were diluted to the working solutions in a simple CaCl_2_ solution (0.1 mM CaCl_2_; pH 5.8), whereas PEG 6000 powders were directly dissolved in a simple CaCl_2_ solution (0.1 mM CaCl_2_; pH 5.8) to 10% and 20% (w/v; working concentration) before use. In addition, the time lengths of all the treatments, including temperature (4 °C and 35 °C), are indicated in the text.

### Quantitative real-time RT-PCR (qRT-PCR) assay

To examine the effects of heavy metal toxicities and other abiotic stresses on the transcriptional expression levels of endogenous *HsCIPKs* in Tibetan Plateau annual wild barley, whole seedlings or roots after treatment were used to isolate the total RNAs using an RNeasy Plant Mini Kit (Qiagen). The first-strand synthesis of cDNA was synthesized with a SuperScript III First-Strand Synthesis System (Invitrogen). The qRT-PCR assay was performed with Thunderbird SYBR qPCR mix (Toyobo) and a StepOnePlus Real-Time PCR System (Applied Biosystems). The reactions were performed in a 20-μL volume containing 10 μL 2 × SYBR qPCR mix (Toyobo), 10 ng cDNA, and 1 μM of each gene-specific primer (Additional file 1: Table S3). The PCR cycles were performed as follows: one cycle of 95 °C for 3 min, 40 cycles of 95 °C for 5 s and 60 °C for 50 s. The resulting data were collected and analyzed using the StepOne Software v2.1. The transcriptional levels were normalized to the housekeeping gene *HvActin* (Additional file 1: Table S3). For each *HsCIPK*, the transcription levels upon stress treatment for different time lengths were presented as relative values of the 0-h time point (mock control; set as 1.0). For statistical analysis (Student’s t-test, two tails; type 2), the transcription levels from three independent experiments at different time-points were compared with those of the mock control.

### Constructs, transformation and molecular identification in rice

To overexpress each *HsCIPK* gene in rice, constructs of *35S::HsCIPKs* were generated individually using PCR, restriction digestion, and ligation with the plant transformation vector pCAMBIA2300S containing a 2× CAMV 35S promoter and a kanamycin-resistant marker (Xiong and Yang 2003). Finally, the resulting constructs were confirmed by sequencing. All the primer sequences for the *HsCIPK* constructs are indicated in Additional file 1: Table S2.

For rice transformation, Nipponbare rice mature embryos were used as the initial materials for callus induction. Briefly, surface-sterilized mature embryos were incubated on agar plates containing N6D media (Ozawa 2009; Toki et al. 2006) for callus induction. Light yellow, compact and hard calluses were used in the Agrobacterium-mediated transformation with *Agrobacterium tumefaciens* EHA105 (Hiei and Komari 2008). Kanamycin-resistant vigorous calluses were recovered on selection solid media containing G418 (150 mg/L; Amresco) due to high-level kanamycin-resistance in the wild-type rice background (Dekeyser et al. 1989) and subsequently transferred to differentiation solid media with G418 (100 mg/L) for green shoot induction. Finally, the roots were induced on solid rooting media with G418 (70 mg/L). After acclimatization, regeneration plantlets (T1 generation) were cultivated in hydroponic conditions.

To determine whether these transgenic plants harboring *35S::HsCIPK* constructs are true overexpression lines, a RT-PCR assay was performed in T1 generation plants. RNA isolation and cDNA synthesis were conducted as described. RT-PCR primers were designed in the *OsCIPK* nonhomologous region of *HsCIPKs* (Additional file 1: Table S4). Rice *OsActin2*, a housekeeping gene, was used as an internal control (Additional file 1: Table S4), whereas the wild-type Nipponbare cDNA sample served as a negative control (NC). Twenty-seven cycles of PCR were used to amplify all the exogenous *HsCIPKs* and the endogenous *OsActin2* in the rice transgenic lines. Homozygous lines for all the transgenic lines overexpressing each *HsCIPK* were recovered in the T3 generation via G418-resistent selection.

### Root length measurement

To determine the roles of the HsCIPKs in the plant response to heavy metal toxicities and other abiotic stresses, primary root growth was used to examine the effect of *HsCIPK* overexpression on the plant growth response to stress treatments. To quantify root elongation, the primary root lengths were individually measured before (0 h) and after 24 h of treatment. To reduce differential physiological effects on root growth before treatment (0 h), root relative elongation rates (RERs; %) were used to evaluate the stress effects on root growth. The RERs were estimated according to the following formula as previously described (Pan et al. 2004; Pan et al. 2011): RER = (RL_T24h_-RL_T0h_)/(RL_M24h_-RL_M0h_) × 100%. RL_T0h_ and RL_T24h_ indicate root lengths (RL; mm) before (0 h) and after the 24-h stress treatment, respectively, whereas RL_M0h_ and RL_M24h_ represent the root lengths before (0 h) and after 24 h of mock treatment, respectively. The quantitative data of 45 seedlings for each treatment from three independent experiments were statistically evaluated using a Student’s *t*-test (two tails; type 2) compared with the nontransgenic regeneration lines (NT; as the wild-type control). Multiple transgenic lines for each construct were examined in the stress treatment and the representative lines are presented.

## Results

### In silico assay of cultivated barley *HvCIPKs*

To obtain the *HvCIPK* cDNA sequences of cultivated barley, we performed an in silico assay to search for the corresponding homologous *CIPKs* in a full-length cDNA library of cultivated barley using the full-length cDNAs of rice *OsCIPK1* to *OsCIPK31* (Kolukisaoglu et al. 2004) as probes and subsequently recovered five rice homologous sequences of *HvCIPKs*, including *CIPK2*, − *9*, − *11*, − *23*, and − *28*.

Next, we used conserved motifs in the kinase and NAF/FISL domains of the OsCIPKs combined with full-length cDNAs of *OsCIPK1* to *OsCIPK31* to BLAST homologous sequences in a nonredundant nucleotide database of cultivated barley and finally obtained eight rice homologous sequences of *HvCIPKs*, including *CIPK5*, − *14*, − *15*, − *17*, − *24*, − *29*, − *30*, and − *31*. Thus, we obtained 13 potential *HvCIPK* cDNA sequences.

### Molecular cloning of the *HsCIPKs* in Tibetan plateau annual wild barley

**Fig. 1:**
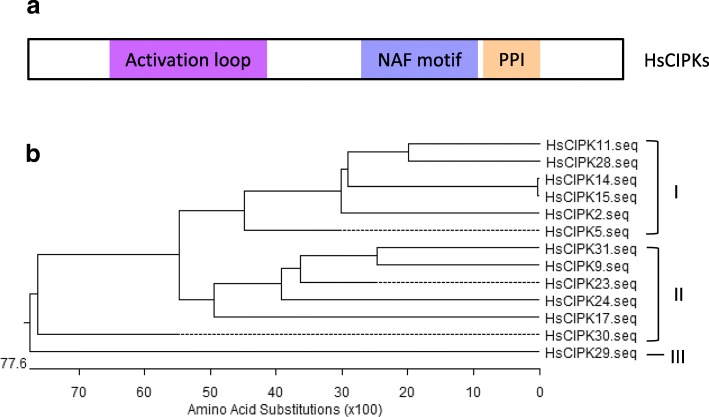
Schematic diagram and sequence analysis of HsCIPKs. **a** Prediction of functional domains in HsCIPKs. **b** Phylogenetic analysis of 13 HsCIPKs

To clone the *HsCIPK* CDS from the Tibetan Plateau annual wild barley, we used these 13 *HvCIPK* CDS sequences, combined with RT-PCR- and sequencing-based approaches, to isolate their homologous sequences from a total cDNA pool of Tibetan Plateau annual wild barley. We finally recovered 13 corresponding full-length CDS sequences of *HsCIPKs*. Previous findings indicated that plant-specific CIPKs possess typical conserved domains, including a kinase domain (activation loop) at the N terminal, a CBL-interacting domain (NAF/FISL motif), and a protein–phosphatase interaction (PPI) domain at the C terminal (Ohta et al. 2003). Analysis of the functional domain prediction confirmed that all of the 13 HsCIPKs indeed contain a conserved activation loop, an NAF/FISL motif, and a PPI domain (Fig. 1a) similar to the rice and Arabidopsis CIPKs (Additional file 1: Figure S2; HsCIPK5 as an example). Phylogenetic analysis of the deduced amino acid sequences showed that these 13 HsCIPKs were evolutionally divided into three branches, including (I) HsCIPK2, − 5, − 11, − 14, − 15, − 28, and − 30, (II) HsCIPK9, − 17, − 23, − 24, and − 31, and (III) HsCIPK29 (Fig. 1b). It is noteworthy that HsCIPK14 has an almost identical sequence to HsCIPK15 except for an additional four amino acids at its 3′-end, similar to the rice and Arabidopsis CIPK14/CIPK15 (Kolukisaoglu et al. 2004), and therefore, only the *HsCIPK14* gene was used in the following studies. In addition, except for *HsCIPK15*, 12 other *HsCIPKs* CDS and their deduced amino acid sequences have been deposited into the GenBank (Additional file 1: Figure S3a).

Next, we analyzed amino acid sequence homologies between the HsCIPKs versus the OsCIPKs and AtCIPKs. The results showed that the HsCIPKs share higher homology with the OsCIPKs (~ 70% to ~ 95%) than the AtCIPKs (~ 46% to ~ 77%) at the amino acid level (Additional file 1: Figure S3b and c). Consistently, phylogenetic tree analysis among the HsCIPKs, OsCIPKs, and AtCIPKs showed that the individual HsCIPKs and corresponding homologous OsCIPKs were located at the identical branches, whereas the AtCIPKs were distributed at the side branches (Additional file 1: Figure S4). These results suggest that the HsCIPKs have a closer genetic relationship to the OsCIPKs than to the AtCIPKs. Thus, we designated the *HsCIPK* numbers with the corresponding homologous *OsCIPK* number.

### Responses of *HsCIPKs* to heavy metal toxicities

**Fig. 2:**
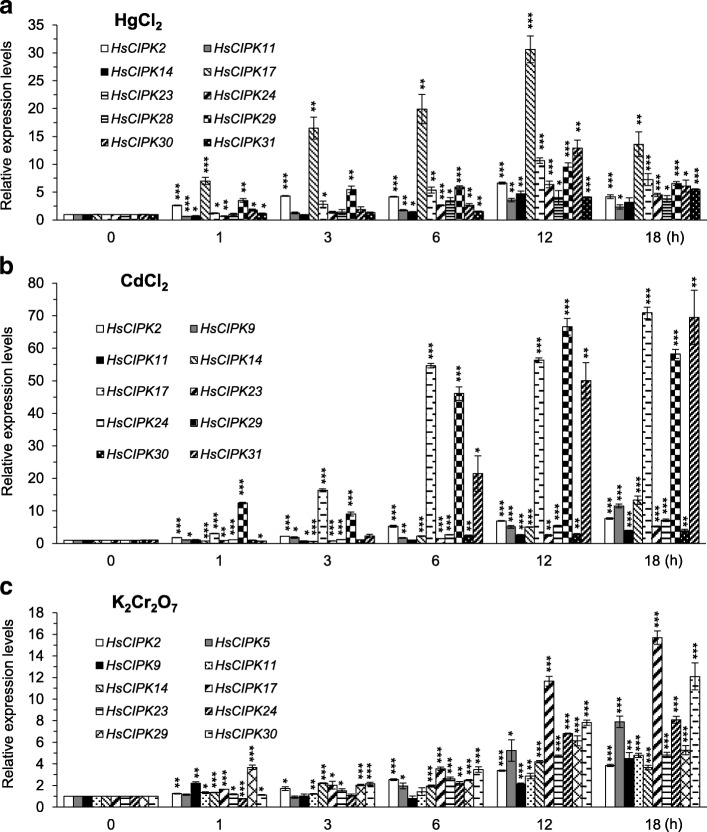
Hg-, Cd-, and Cr-induced expression patterns of *HsCIPKs*. **a-c** Relative expression levels of *HsCIPKs* in four-day-old wild barley seedlings treated for different time lengths (0, 1, 3, 6, 12, and 18 h) with 20 μM HgCl_2_ (**a**), 20 μM CdCl_2_ (**b**), and 0.5 mM K_2_Cr_2_O_7_ (**c**), respectively. Values shown are means ± SD. Single, double, and triple asterisks indicate *P* < 0.01, 0.001, and 0.0001, respectively (*t* test; compared to the corresponding 0-time point mock control). The expression level of the 0-time point mock control for each *HsCIPK* was set as 1.0

**Fig. 3:**
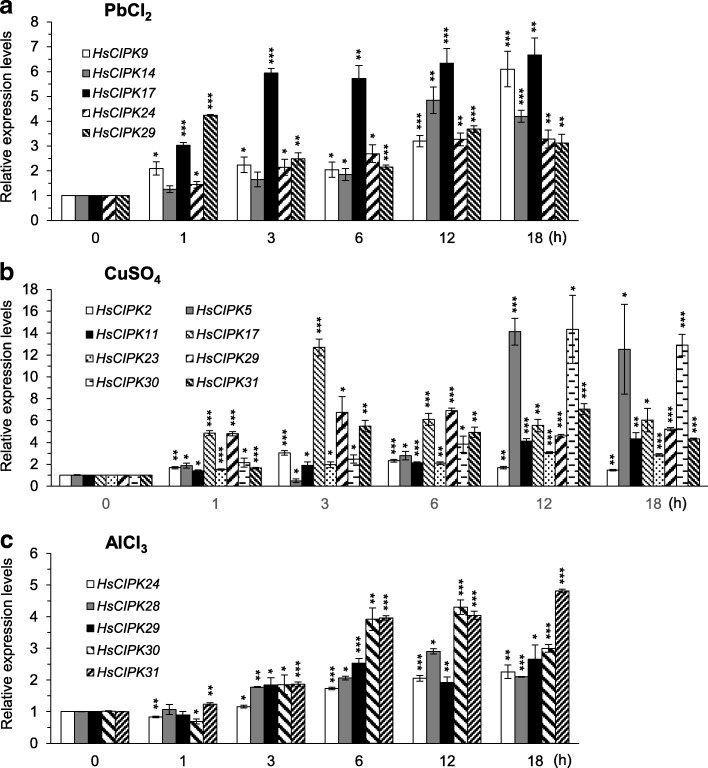
Pb-, Cu-, and Al-induced expression patterns of *HsCIPKs*. **a-c** Relative expression levels of *HsCIPKs* in four-day-old wild barley seedlings treated for different time lengths (0, 1, 3, 6, 12, and 18 h) with 20 μM PbCl_2_ (**a**), 1 mM CuSO_4_ (**b**), and 20 μM AlCl_3_ (**c**), respectively. Values shown are means ± SD. Single, double, and triple asterisks indicate P < 0.01, 0.001, and 0.0001, respectively (*t* test; compared to the corresponding 0-time point mock control). The expression level of the 0-time point mock control for each *HsCIPK* was set as 1.0

It is well known that plant CIPKs function in the regulation of various abiotic and biotic responses (Guo et al. 2001; de la Torre et al. 2013). However, whether CIPKs are involved in the plant response to heavy metal toxicities is largely unknown. To this end, we performed a time-course qRT-PCR analysis to examine whether heavy metal toxicities influence the transcriptional expression levels of the endogenous *HsCIPKs* in the Tibetan Plateau annual wild barley seedlings treated with HgCl_2_ (20 μM), CdCl_2_ (20 μM), K_2_Cr_2_O_7_ (0.5 mM), PbCl_2_ (20 μM), and CuSO_4_ (1 mM), respectively. As shown in Fig. 2a, upon seedling exposure to HgCl_2_ treatment, the transcriptional levels of 10 genes (*HsCIPK2, 11, 14*, *17, 23*, *24, 28*, *29, 30*, and *31*) were dramatically elevated relative to the mock control (0 h time point). Consistently, the expression levels of 10 genes (*HsCIPK2, 9*, *11, 14*, *17, 23*, *24, 29, 30*, and *31*) in the CdCl_2_ treatment (Fig. 2b), 10 genes (*HsCIPK2*, *5*, *9*, *11, 14*, *17*, *23*, *24, 29*, and *30*) in the K_2_Cr_2_O_7_ treatment (Fig. 2c), five genes (*HsCIPK9, 14, 17, 24,* and *29*) in the PbCl_2_ treatment (Fig. 3a), and eight genes (*HsCIPK2*, *5*, *11*, *17*, *23*, *29*, *30*, and *31*) in the CuSO_4_ treatment (Fig. 3b) were significantly increased compared to those in the corresponding mock control (0 h time point). In addition, the maximum induction levels of all 12 endogenous *HsCIPKs* during each heavy metal treatment are briefly summarized in Additional file 1: Table S5, whereas these *HsCIPKs* that were transcriptionally three times higher than the mock control are presented in Figs. 2 and 3. These results suggest that the *HsCIPKs* respond transcriptionally to heavy metal toxicities.

### Responses of *HsCIPKs* to other abiotic stresses

**Fig. 4:**
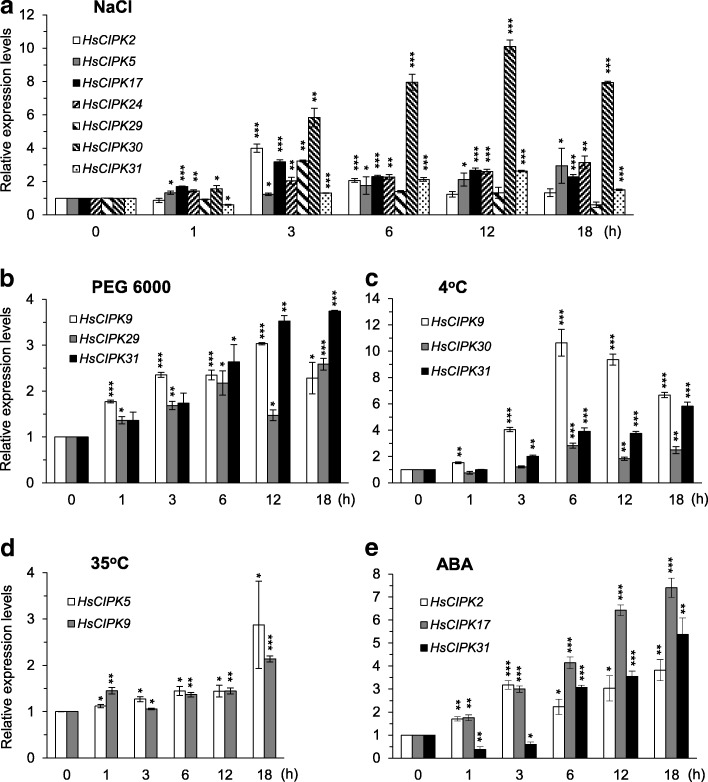
Salt-, drought-, temperature-, and ABA-induced expression patterns of *HsCIPKs*. **a-e** Relative expression levels of *HsCIPKs* in four-day-old wild barley seedlings treated for different time lengths (0, 1, 3, 6, 12, and 18 h) with 400 mM NaCl (**a**), 20% PEG 6000 (**b**), 4 °C (**c**), 35 °C (**d**), and 20 μM ABA (**e**), respectively. Values shown are means ± SD. Single, double, and triple asterisks indicate P < 0.01, 0.001, and 0.0001, respectively (*t* test; compared to the corresponding 0-time point mock control). The expression level of the 0-time point mock control for each *HsCIPK* was set as 1.0

In additional to heavy metal toxicities, we also examined the effects of other abiotic stresses on the transcriptional expression levels of these 12 *HsCIPKs* in the Tibetan Plateau annual wild barley seedlings, including treatments with NaCl (400 mM), polyethylene glycol 6000 (PEG 6000; 20%; artificial drought), AlCl_3_ (20 μM), low and high temperature (4 °C and 35 °C), and ABA (20 μM), respectively. Time-course analysis showed that the transcriptional levels of seven genes (*HsCIPK2, 5, 17, 24, 29, 30*, and *31*) in the NaCl treatment (Fig. 4a), three genes (*HsCIPK9*, *29*, and *31*) in the PEG6000 treatment (Fig. 4b), five genes (*HsCIPK24*, *28*, *29*, *30*, and *31*) in the AlCl_3_ treatment (Fig.3c), three genes (*HsCIPK9, 30* and *31*) in the 4 °C treatment (Fig. 4c), two genes (*HsCIPK5* and *9*) in the 35 °C treatment (Fig. 4d), and three genes (*HsCIPK2*, *17*, and *31*) in the ABA treatment (Fig. 4e) were significantly elevated relative to the corresponding mock control (0 h time point). Furthermore, the maximum expression levels of all 12 endogenous *HsCIPKs* during salt, PEG, AlCl_3_, temperature (4 °C and 35 °C), and ABA treatments are briefly summarized in Additional file 1: Table S6, whereas their induction levels, which were two times higher than the mock control are presented in Figs. 3c and 4. These results further confirmed the involvement of *HsCIPKs* in multiple abiotic stress responses.

### Generation of rice transgenic lines overexpressing *HsCIPKs*

**Fig. 5:**
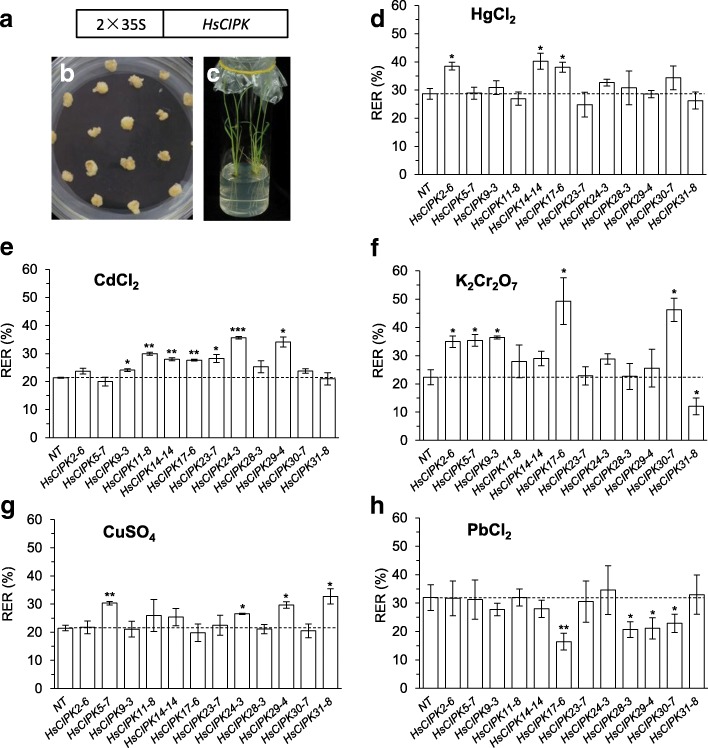
Effects of heavy metal toxicities on root growth in rice seedlings overexpressing *HsCIPKs*. **a-c** Construct for *HsCIPK* overexpression (**a**), callus induction in rice mature embryos (**b**), and transgenic plants (**c**). d-h Root relative elongation rates (%; RER) of four-day-old rice seedlings overexpressing *HsCIPKs* treated for 24 h with 0.5 μM HgCl_2_ (**d**), 5 μM CdCl_2_ (**e**), 5 μM K_2_Cr_2_O_7_ (**f**), 0.25 μM CuSO_4_ (**g**), and 5 μM PbCl_2_ (**h**), respectively. Values shown are means ± SD. Single, double, and triple asterisks indicate *P* < 0.05, 0.01, and 0.001, respectively (*t* test; compared to the wild-type control NT seedlings). NT, nontransgenic regeneration lines

To provide further evidence for the roles of HsCIPKs in the plant response to heavy metal toxicities and other abiotic stresses, rice transgenic lines overexpressing individual *HsCIPKs* under the control of the CAMV 35S promoter were generated in the wild-type rice cultivar Nipponbare (*Oryza sativa* L. ssp. japonica) (Fig. 5a-c), which is a good transformation system and additionally possesses a closer genetic relationship to the wild barley than Arabidopsis at the CIPK levels (Additional file 1: Figsure S3 and S4). A RT-PCR assay with *HsCIPK*-specific PCR primers was used to examine the overexpression levels of the exogenous *HsCIPK* genes in the T1 generation plants. As shown in Additional file 1: Figure S5, multiple overexpression transgenic lines for each *HsCIPK* construct were recovered. In the T3 generation, homozygous transgenic lines with no further segregation were recovered via G418-resistant selection.

### Functional analysis of *HsCIPK* overexpression in response to heavy metal toxicities

In plants, heavy metal toxicity-induced symptoms include the inhibition of seed germination and root elongation, wilting and stunted plant growth, chlorosis, leaf rolling and necrosis, and senescence, as well as low biomass (DalCorso et al. 2013), among which root elongation inhibition is an early symptom of heavy metal-induced responses. Thus, root elongation has been widely used as a sensitive indicator for plant responses to heavy metal toxicities (Wong and Bradshaw 1982) and other abiotic stresses (Llugany et al. 1995; Ishikawa et al. 1998). In this study, the relative elongation rate (RER; %) of the primary root was used to evaluate the root growth response to heavy metal toxicities. Heavy metal treatment experiments with different concentrations in four-day-old wild-type Nipponbare seedlings revealed that 0.5 μM HgCl_2_, 5 μM CdCl_2_, 5 μM K_2_Cr_2_O_7_, 0.25 μM CuSO_4_, and 5 μM PbCl_2_ were suitable to examine the effects of *HsCIPK* overexpression on root growth response to heavy metal toxicities (Additional file 1: Figure S1). Upon transgenic seedling exposure to HgCl_2_ treatment for 24 h, the overexpression of *HsCIPK2*, *HsCIPK14*, and *HsCIPK17* significantly enhanced root growth relative to the corresponding wild-type controls (nontransgenic regeneration lines; NT) (Fig. 5d). Similarly, transgenic lines individually overexpressing seven *HsCIPKs* (*HsCIPK9*, *11*, *14*, *17*, *23*, *24*, and *29*) in the CdCl_2_ treatment (Fig. 5e), five *HsCIPKs* (*HsCIPK2*, *5*, *9*, *17*, and *30*) in the K_2_Cr_2_O_7_ treatment (Fig. 5f), and four *HsCIPKs* (*HsCIPK5*, *24*, *29*, and *31*) in the CuSO_4_ treatment (Fig. 5g) displayed a significant elevation in root growth relative to the corresponding NT. In the PbCl_2_ treatment, surprisingly, no transgenic lines displayed enhanced effects in root growth relative to the NT (Fig. 5h). In contrast, the transgenic lines overexpressing *HsCIPK31* in the K_2_Cr_2_O_7_ treatment (Fig. 5f) and *HsCIPK17*, *28*, *29*, and *30* in the PbCl_2_ treatment (Fig. 5h), respectively, exhibited a significant reduction in root growth compared to the corresponding NT. The responses of root growth to these heavy metal toxicities in the transgenic lines of all 12 *HsCIPKs* are summarized in Additional file 1: Table S7. Taken together, these results suggest that multiple *HsCIPKs* are involved in the plant response to heavy metal toxicities.

### Functional analysis of *HsCIPK* overexpression in response to other abiotic stresses

**Fig. 6:**
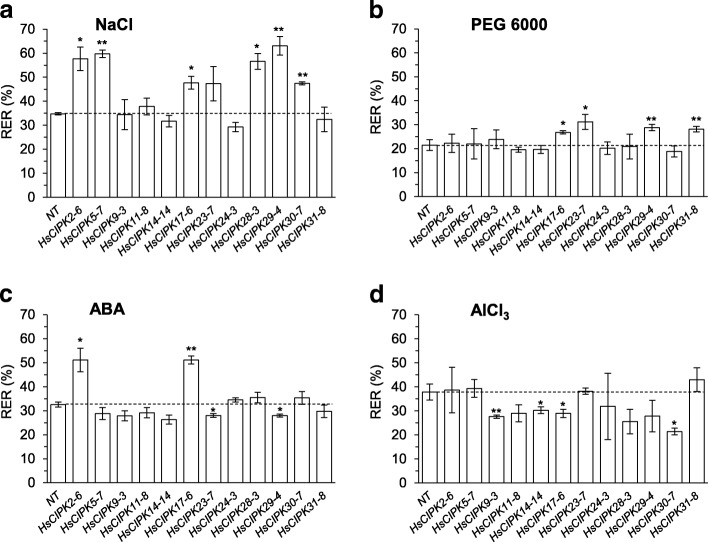
Effects of salt, drought, ABA, and Al treatments on root growth in rice seedlings overexpressing *HsCIPKs*. **a-d** Root relative elongation rates (%; RER) of four-day-old rice seedlings overexpressing *HsCIPKs* treated for 24 h with 50 mM NaCl (**a**), 10% PEG6000 (**b**), 1 μM ABA (**c**), and 50 μM AlCl_3_ (**d**), respectively. Values shown are means ± SD. Single and double asterisks indicate P < 0.05 and 0.01, respectively (*t* test; compared to the wild-type control NT seedlings). NT, nontransgenic regeneration lines

Next, to test whether the overexpression of *HsCIPKs* enhances plant tolerance to other abiotic stresses, we examined root growth of four-day-old transgenic rice seedlings treated for 24 h with salt (50 mM NaCl), drought (10% PEG6000), ABA (1 μM), and AlCl_3_ (50 μM) (Additional file 1: Figure S1), respectively. As shown in Fig. 6a-c, upon transgenic seedling exposure to NaCl, PEG6000, and ABA treatments, respectively, the overexpression lines for six *HsCIPKs* (*HsCIPK2*, *5*, *17*, *28*, *29*, and *30*) in the NaCl treatment, four *HsCIPKs* (*HsCIPK17*, *23*, *29*, and *31*) in PEG 6000 treatment, and two *HsCIPKs* (*HsCIPK2* and *17*) in the ABA treatment displayed a significantly enhanced effect on root growth relative to the corresponding NT, indicating a positive role of these *HsCIPKs* in plant tolerance to salt and drought stresses and ABA treatment. However, in the AlCl_3_ treatment, no transgenic lines showed an enhanced effect on root growth relative to the NT (Fig. 6d). In contrast, the transgenic lines overexpressing two *HsCIPKs* (*HsCIPK23* and *29*) in the ABA treatment (Fig. 6c) and four *HsCIPKs (HsCIPK9*, *14*, *17*, and *30*) in the AlCl_3_ treatment (Fig. 6d) exhibited an inhibitory effect on root growth relative to the corresponding NT, indicating a negative role of these *HsCIPKs* in the plant response to ABA and AlCl_3_ stresses. The responses of root growth to these abiotic stresses in the transgenic lines of all 12 *HsCIPKs* are summarized in Additional file 1: Table S7. These results further confirmed the roles of *HsCIPKs* in the plant response to salt and drought stresses.

## Discussion

Previous studies have demonstrated that plant-specific CIPKs function in plant responses to various abiotic and biotic stresses, including salt, drought, low and high temperature, wounding, low oxygen, and pathogen infection (Shen et al. 2014; Yu et al. 2014). However, the evidence for whether *CIPKs* are involved in the plant esponse to heavy metal toxicities currently remains lacking. In this study, we used a*n* in silico assay and a molecular cloning strategy to isolate 12 *HsCIPKs* from Tibetan Plateau annual wild barley and subsequently examined the heavy metal toxicity-induced expression patterns of 12 endogenous *HsCIPK* genes and determined the role of their overexpression in the rice response to multiple heavy metal toxicities and other abiotic stresses.

### Heavy metal toxicities induce transcriptional expression of multiple *HsCIPKs*

Our time-course qRT-PCR analysis (Figs. 2, 3 and 4; Additional file 1: Tables S5 and S6) revealed that all of the 12 endogenous *HsCIPKs* examined can be induced by multiple heavy metal toxicities, including Hg, Cd, Cr, Pb, and Cu, and in addition, nine *HsCIPKs* (except for *HsCIPK11*, *14*, and *23*) are also induced by multiple other abiotic stresses, including salt, drought, cold, heat, and Al stresses, and ABA treatment. Heavy metal treatment experiments (Figs. 2 and 3; Additional file 1: Table S5) showed that *HsCIPK17* and *29* can be highly induced by all five heavy metal toxicities examined, whereas *HsCIPK2*, *11*, *14*, *23*, *24*, and *30* are highly induced by four heavy metal toxicities. In addition, *HsCIPK9* and *31* are also highly induced by three heavy metal toxicities. In addition to the response to heavy metal toxicities, *HsCIPKs* also respond to other abiotic stresses, including salt, drought, temperature, and Al stresses. Of note, the transcriptional induction levels corresponding to these stresses (Fig. 4; Additional file 1: Table S6) are relatively low compared to those corresponding to heavy metals (Figs. 2 and 3; Additional file 1: Table S5). These data suggest that multiple *HsCIPKs* are involved in the plant response to heavy metal toxicities and other abiotic stresses.

Previous studies have shown that wheat *TaCIPK14* and Arabidopsis *AtCIPK14* are upregulated by treatments with salt, PEG, and ABA (Deng et al. 2013; Yan et al. 2014). In contrast, our treatment experiments (Additional file 1: Tables S5 and S6; Figs. 2 and 3) revealed that *HsCIPK14* is downregulated by salt, PEG, heat, and ABA treatments but upregulated by multiple heavy metal toxicities, including Hg, Cd, Cr, and Pb. Similarly, *Brachypodium distachyon BdCIPK31* was found to be downregulated by salt, PEG, H_2_O_2_, and ABA treatments (Luo et al. 2017), whereas *HsCIPK31* is upregulated by not only salt, PEG, ABA, Al, and cold treatments but also multiple heavy metal toxicities, including Hg, Cd, and Cu (Figs. 2, 3 and 4; Additional file 1: Tables S5 and S6). These different responses clearly reflect the functional divergence of the *CIPK* paralog genes in distinct plant species in response to various abiotic stresses during long-term evolution.

### Overexpression of the *HsCIPKs* enhances plant tolerance to heavy metal toxicities

The overexpression experiment in rice (Figs. 5 and 6; Additional file 1: Table S7) showed that the individual overexpression of 11 *HsCIPKs* (except for *HsCIPK28*) promotes root growth tolerance to multiple heavy metal toxicities (Hg, Cd, Cr, and Cu), and furthermore, plants individually overexpressing 8 *HsCIPKs* (except for *HsCIPK9*, *11*, *14*, and *24*) display enhanced tolerance of root growth to salt, drought, and ABA treatments, further confirming the involvement of the *HsCIPKs* in the plant response to heavy metal toxicities and other abiotic stresses, including salt and drought. However, we cannot rule out the possibility that the transgenic lines of some *HsCIPKs* with no positive role in root growth tolerance to most of stress treatments are due to their inadequate expression levels, for example, *HsCIPK11* and *HsCIPK23* (Additional file 1: Figure S5). In addition, PbCl_2_ and AlCl_3_ treatments in wild barley significantly induce the transcriptional expression of multiple *HsCIPKs* (*9*, *14*, *17*, *24*, *28*, *29*, *30*, and *31*; Fig. 3; Additional file 1: Tables S5 and S6), but the overexpression of *HsCIPKs* (*9*, *14*, *17*, *28*, *29*, and *30*) in rice inhibits the root growth tolerance to Pb and Al toxicities (Figs. 5 and 6; Additional file 1: Table S7). Similarly, the overexpression of *HsCIPK31* in rice inhibits the root growth tolerance to Cr toxicity, which does not visibly induce *HsCIPK31* transcriptional expression (Fig. 5; Additional file 1: Tables S6 and S7). Although Al and heavy metal toxicities affect plant root growth differentially, our results clearly indicate that these HsCIPKs act as a negative regulator in the plant tolerance to Pb, Al, and Cr toxicities. This finding is similar to the previous studies showing that ABA treatment induces endogenous *BnCIPK6* and *AtCIPK6* expression transcriptionally, although their overexpression in *Arabidopsis* enhances the sensitivity to the ABA treatment (Chen et al. 2012; Chen et al. 2013), and that the loss of *AtCIPK5* function mutants display an increased resistance response to pathogens (Meteignier et al. 2017), and therefore Bn/AtCIPK6 and AtCIPK5 are a negative regulator for plant tolerance to ABA treatment and pathogen attack, respectively. It is likely that during the plant response to Pb, Al, and Cr toxicities, these HsCIPKs with a negative role may exert feedback regulation on the positive functions of other HsCIPKs/CBLs and/or other calcium sensors (CaMs, CMLs, and CDPKs). This hypothesis will require additional study. Fortunately, a high-quality reference genome assembly for cultivated barley has been recently published (Mascher et al. 2017) and thereby will accelerate the functional identification of all *HsCIPK* family members.

It is widely acknowledged that multiple CIPKs in distinct plant species redundantly function in salt, drought, and ABA stress responses (Luan 2009; Weinl and Kudla 2009; Shen et al. 2014; Yu et al. 2014). Consistently, our findings (Figs. 5 and 6; Additional file 1: Table S7) reveal that the same single *HsCIPK* is involved in the response to multiple distinct heavy metal toxicities, whereas multiple *HsCIPKs* function in the response to the same single heavy metal toxicity, indicating that plant-specific *CIPKs* are functionally redundant in the regulation of the plant response to heavy metal toxicities. The identification of knockout or knockdown mutants for *HsCIPKs* will provide genetic evidence for HsCIPK function in heavy metal toxicities.

The traditional remediation strategies for heavy metals-contaminated soils primarily depend on physical and chemical methods and are expensive and relatively ineffective due to large-scale contamination farmlands and high costs. Thus, the best approaches are to genetically engineer crops with high resistance to heavy metal toxicities (Cao et al. 2014). Rice, as a primary food cereal in the world, is one of the major sources of heavy metal intakes for humans in inland China (Zhang et al. 2010), and therefore, developing rice cultivars with a higher tolerance to multiple heavy metal toxicities is critical for heavy metal-polluted soils that usually contain multiple heavy metal toxicities. Intriguingly, our results (Figs. 5 and 6; Additional file 1: Table S7) showed that the overexpression of the same single *HsCIPK* enhances rice growth tolerance to multiple heavy metal toxicities, indicating that these identified *HsCIPKs* (*2*, *5*, *9*, *14*, *17*, *24*, and *29*) are important for genetically engineering crops with multiple heavy metal tolerances, which will have a broad application in heavy metal-contaminated soils. However, the mode of action by which HsCIPKs enhance plant tolerance to heavy metal toxicities via modulating heavy metal excretion and/or chelation mechanisms or scavenging heavy metal-induced ROS remain to be experimentally elucidated in future studies.

## Conclusions

Using an in silico assay and a molecular cloning strategy to isolate 12 *HsCIPKs* in Tibetan Plateau annual wild barley enabled us to the effects of heavy metal toxicities on the transcriptional expression patterns of 12 endogenous *HsCIPK* genes and evaluate their functional roles in the response to multiple heavy metal toxicities and other abiotic stresses in rice. Multiple *HsCIPKs* were found to be involved in the response of plants to heavy metal toxicities, including Hg, Cd, Cr, Pb, and Cu and other abiotic stresses, including salt, drought, Al, low and high temperature, and ABA. The ectopic overexpression of *HsCIPK2*, *5*, *9*, *14*, *17*, *24*, and *29* in Nipponbare rice (*Oryza sativa*) enhanced the tolerance of rice root growth to multiple heavy metal toxicities, whereas the overexpression of *HsCIPK2*, *5*, *17*, 23, *24*, 28, *29*, 30, and 31 promoted root growth tolerance to salt and/or drought stresses. These results suggest that plant-specific CIPKs function in heavy metal toxicities and these *HsCIPKs* examined will have a broad application in genetically engineered rice and other crops with tolerance to heavy metal toxicities and other abiotic stresses.

## Additional file

Additional file 1: **Figure S1.** Effects of abiotic stresses on root elongation in rice. **Figure S2.** Alignment analysis of amino acid sequences among HsCIPK5, OsCIPK5, and AtCIPK5. **Figure S3.** GenBank accession numbers and homology analysis of HsCIPKs. **Figure S4.** Phylogenetic analysis of HsCIPKs, OsCIPKs, and AtCIPKs. **Figure S5.** Molecular identification of *HsCIPK* overexpression in rice. **Table S1.** Accession numbers of rice and *Arabidopsis CIPK* cDNA in NCBI database. **Table S2.** Primer sequences for *HsCIPKs* cloning. **Table S3.** Primer sequences for qRT-PCR assay in Tibetan Plateau annual wild barley. **Table S4.** Primer sequences for RT-PCR Assay in rice transgenic lines. **Table S5.** Summary of endogenous *HsCIPK* responses to heavy metal toxicities. **Table S6.** Summary of endogenous *HsCIPK* responses to other abiotic stresses. **Table S7.** Summary of root growth tolerance to abiotic stresses in rice transgenic lines overexpressing *HsCIPKs.* (PDF 800 kb)

## Abbreviations

ABA: Abscisic acid
Al: Aluminum
Ca^2+^: Calcium
CBL: Calcineurin B-like protein
Cd: Cadmium
CDS: Coding sequence
CIPK: CBL-interacting protein kinase
Cr: Chromium
Cu: Copper
Hg: Mercury
NAF/FISL: Conserved amino acid motif NAF/FISL
NC: Negative control
NT: Nontransgenic regeneration lines
Pb: Lead
PEG 6000: Polyethylene glycol 6000
PPI: Protein–phosphatase interaction domain
qRT-PCR: Quantitative Real-Time PCR
RER: Relative elongation rates
RT-PCR: Reverse transcription-polymerase chain reaction
