# Supplementary material for: Overexpression of the Tibetan Plateau annual wild barley (Hordeum spontaneum) HsCIPKs enhances rice tolerance to heavy metal toxicities and other abiotic stresses
Source: Rice (N Y). 2018 Sep 12;11:51. doi: 10.1186/s12284-018-0242-1 (PMC6135728; doi:10.1186/s12284-018-0242-1)
Supplement: Supplementary file 1 — Figure S1. Effects of abiotic stresses on root elongation in rice. Figure S2. Alignment analysis of amino acid sequences among HsCIPK5, OsCIPK5, and AtCIPK5. Figure S3. GenBank accession numbers and homology analysis of HsCIPKs. Figure S4. Phylogenetic analysis of HsCIPKs, OsCIPKs, and AtCIPKs. Figure S5. Molecular identification of HsCIPK overexpression in rice. Table S1. Accession numbers of rice and Arabidopsis CIPK cDNA in NCBI database. Table S2. Primer sequences for HsCIPKs cloning. Table S3. Primer sequences for qRT-PCR assay in Tibetan Plateau annual wild barley. Table S4. Primer sequences for RT-PCR Assay in rice transgenic lines. Table S5. Summary of endogenous HsCIPK responses to heavy metal toxicities. Table S6. Summary of endogenous HsCIPK responses to other abiotic stresses. Table S7. Summary of root growth tolerance to abiotic stresses in rice transgenic lines overexpressing HsCIPKs. (PDF 800 kb) [file 12284_2018_242_MOESM1_ESM.pdf]

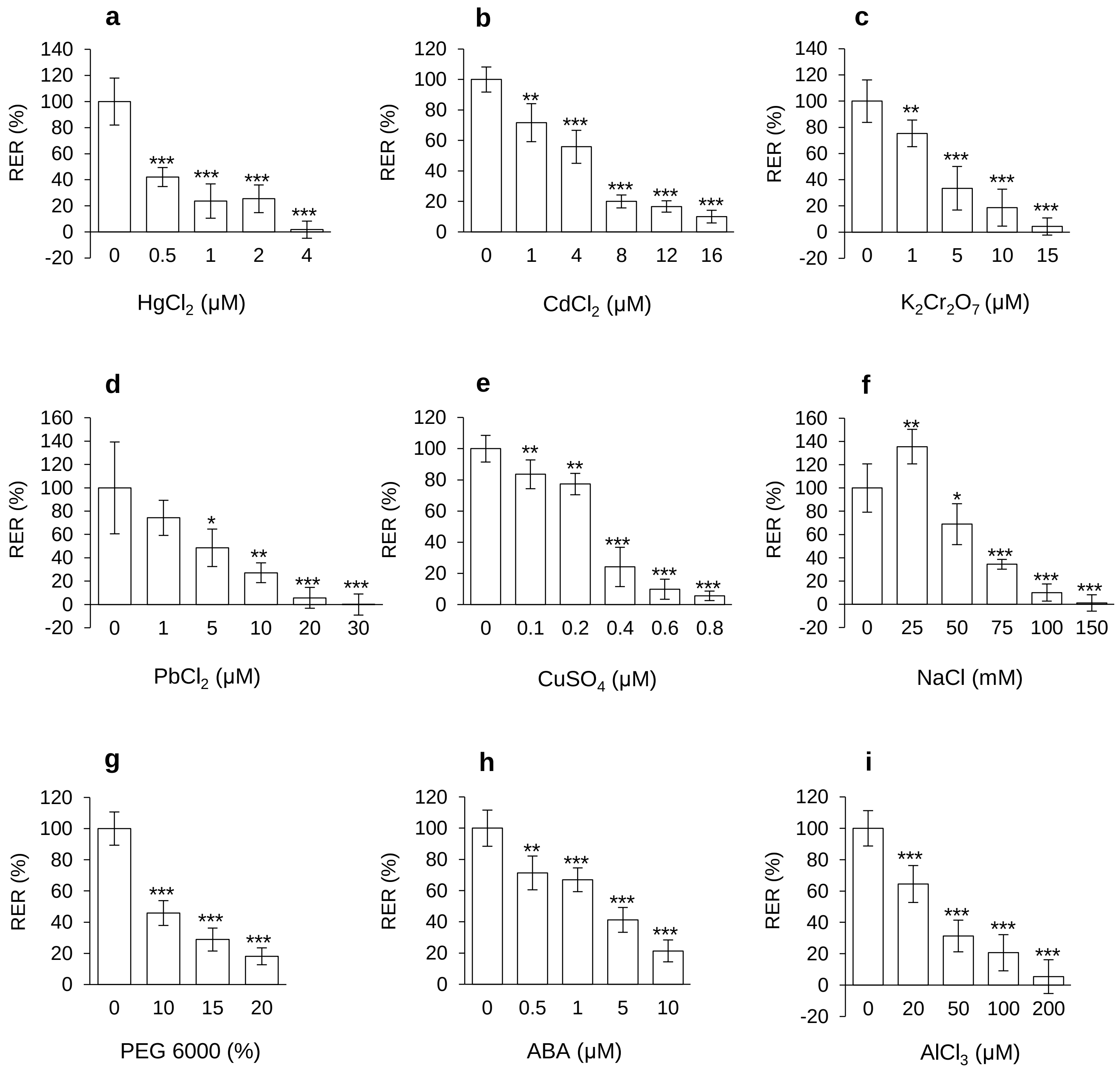

Figure S1. Effects of heavy metal toxicities and other abiotic stresses on root growth in rice.

a-i Four-day-old wild-type rice seedlings were treated for 24 h with different concentrations (as indicated) of heavy metal salts, including $HgCl_2$ (**a**), $CdCl_2$ (**b**), $K_2Cr_2O_7$ (**c**), $PbCl_2$ (**d**), and $CuSO_4$ (**e**), and other abiotic stresses, including NaCl (**f**), PEG 6000 (**g**), ABA (**h**), and $AlCl_3$ (**i**). The effects of these abiotic stresses examined on root elongation were presented as root relative elongation rates (%; RER) compared to the corresponding mock control (100%). Values shown are means ± SD. Single, double, and triple asterisks indicate $P < 0.01$, 0.001, and 0.0001, respectively (*t* test; compared to the corresponding mock control).

```
AtCIPK5: MEEERRVLFGKYEMGRLLGKGTFAKVYYGKEIIGGECVAIKVINKDQVMKRPGMMEQIKREISIMKLV :  68
OsCIPK5: MEKKASILMNRYELGRMLGQGTFAKVYHARNLASNQSVAIKVIDKEKVL-RVGMIDQIKREISIMRLV :  67
HsCIPK5: MERKSTILMNRYELGRMLGQGTFAKVYHARSLATNQSVAIKVIDKEKVL-RVGMIDQIKREISIMRLV :  67

AtCIPK5: RHPNIVELKEVMATKTKIFFVMEFVKGGELFCKISKGKLHEDAARRYFQQLISAVDYCHSRGVSHRDL : 136
OsCIPK5: RHPNIVQLHEVMASKSKIYFAMEYVRGGELFSRVARGRLKEDAARKYFQQLIGAVDFCHSRGVYHRDL : 135
HsCIPK5: RHPNIVQLHEVMASKSKIYFAMEYVRGGELFARVAKGRLKEDAARKYFQQLIGAVDFCHSRDVYHRDL : 135

                             Activation Loop
AtCIPK5: KPENLLLDENGDLKISDFGLSALPEQILQDGLLHTQCGTPAYVAPEVLKKKGYDGAKADIWSCGVVLY : 204
OsCIPK5: KPENLLVDENGNLKVSDFGLSAFKECQKQDGLLHTTCGTPAYVAPEIINKRGYDGAKADIWSCGVILF : 203
HsCIPK5: KPENLLVDEHGNLKVSDFGLSALKECQKQDGLLHTTCGTPAYVAPEIINKKGYDGEKADIWSCGVILF : 203

AtCIPK5: VLLAGCLPFQDENLMNMYRKIFRADFEFPPWFSPEARRLISKLLVVDPDRRISIPAIMRTPWLRKNFT : 272
OsCIPK5: VLLAGYLPFHDSNLMEMYRKISKGDVKFPQWFTTDVRRLLSRLLDPNPNIRITVEKLVEHPWFKKGYK : 271
HsCIPK5: VLLAGYLPFQDSNLMEMYRKISKGDVRYPQWFSSDARKLLCRLLDPNPNTRITMDKLIEHPWFKKGYK : 271

                                                    NAF/FISL Motif
AtCIPK5: PPLAFKIDEPICSQSSK------NNEEEEEDG-DCENQTEPISPKFFNAFEFISSMSSGFDLSSLFES : 333
OsCIPK5: PAV--MLSQPNESNNLKDVHTAFSADHKDNEG-KAKEPASSLKPVSLNAFD-IISLSKGFDLSGLFEN : 335
HsCIPK5: PAV--MLGTPRASKDLNDVQAAFSTDHKDGEGSRAEQPNSPLKPASLNAFD-IISLSKGFDLSGLFEK : 336

AtCIPK5: KR--KVQSVFTSRSSATEVMEKIETVTKEMNMKVKRTKDFKVKMEGKTEGRKGRLSMTAEVFEVAPEI : 399
OsCIPK5: DKEQKADSRFMTQKPASAIVSKLEQIAETESFKVKKQDGL-VKLQGSKEGRKGQLAIDAEIFEVTPSF : 402
HsCIPK5: DQEQKSNSRFMTQKPASAIVSKLEQIAETESFKVKKQDGL-VTLQGSKEGRKGQLAIDAEIFEVTPSF : 403
                          PPI

AtCIPK5: SVVEFCKSAGDTLEYDRLYEEEVRPALNDIVWSWHGDNNN--TSSEDC---------------- : 445
OsCIPK5: FVVEVKKSAGDTLEYEKFCNKGLRPSLRDI--CWDGQSEH--PSLAQSSTLTQSSKSISRHAI- : 461
HsCIPK5: YVVEVKKSAGDTLEYERFCKKGLRPSLKDI--CWNGPSEEKLPLVSESVPPTPSSKSTKRNVI- : 464
```

Figure S2. Alignment analysis of amino acid sequences among HsCIPK5, OsCIPK5, and AtCIPK5.

a

Gene	Accession number	Gene	Accession number
HsCIPK2	KP638475	*HsCIPK23*	JN655678
HsCIPK5	KT274758	*HsCIPK24*	JN655679
HsCIPK9	JN655674	*HsCIPK28*	KT274759
HsCIPK11	JN655675	*HsCIPK29*	JN655680
HsCIPK14	JN655676	*HsCIPK30*	JN655681
HsCIPK17	JN655677	*HsCIPK31*	JN655682

b

	OsCIPK2	OsCIPK5	OsCIPK9	OsCIPK11	OsCIPK14	OsCIPK15	OsCIPK17	OsCIPK23	OsCIPK24	OsCIPK28	OsCIPK29	OsCIPK30	OsCIPK31
HsCIPK2	**86.2**	62.0	51.5	59.3	59.9	59.4	41.2	55.1	47.1	58.8	40.1	49.8	52.5
HsCIPK5	61.8	**85.9**	51.3	54.6	58.2	58.2	44.2	56.5	49.2	55.4	40.2	54.3	52.5
HsCIPK9	51.6	51.5	**90.3**	49.1	46.2	46.3	47.9	67.1	55.0	46.6	37.2	47.2	62.6
HsCIPK11	61.8	54.2	43.8	**80.7**	58.7	58.2	39.7	50.2	43.7	72.2	40.9	49.7	48.9
HsCIPK14	59.4	56.7	46.3	60.5	**82.7**	82.8	41.7	49.4	43.5	59.4	39.4	49.1	49.2
HsCIPK15	59.4	57.2	47.1	60.8	83.0	**83.0**	42.3	50.1	44.2	59.5	39.6	49.1	49.6
HsCIPK17	41.1	42.4	44.7	39.6	40.4	40.4	**80.8**	47.6	44.0	39.3	34.5	38.3	45.9
HsCIPK23	55.6	54.8	65.5	51.6	50.9	50.8	46.8	**94.7**	56.6	49.5	39.6	46.9	63.3
HsCIPK24	46.2	47.7	53.8	44.8	44.1	44.6	43.5	56.4	**89.1**	44.9	39.2	43.8	54.2
HsCIPK28	58.0	53.0	42.6	67.4	59.5	59.2	39.3	49.0	45.5	**73.8**	42.3	48.2	47.2
HsCIPK29	42.0	40.2	35.7	40.8	42.3	42.1	35.2	38.0	36.7	40.5	**70.0**	40.6	36.6
HsCIPK30	52.8	54.8	49.3	47.4	50.5	50.1	37.9	48.5	43.0	47.6	38.8	**77.3**	49.5
HsCIPK31	54.2	52.0	64.7	50.1	49.3	49.2	45.9	63.3	52.5	47.4	35.5	48.4	**92.0**

c

	AtCIPK2	AtCIPK5	AtCIPK9	AtCIPK11	AtCIPK14	AtCIPK15	AtCIPK17	AtCIPK23	AtCIPK24
HsCIPK2	**61.0**	55.1	54.5	48.6	48.9	61.6	42.9	55.3	47.2
HsCIPK5	59.3	**52.5**	51.4	49.3	48.1	58.3	43.3	54.5	48.2
HsCIPK9	46.4	50.8	**72.1**	44.3	44.1	52.3	47.9	67.8	54.5
HsCIPK11	61.8	54.2	50.1	**48.1**	45.1	57.2	40.2	48.6	46.2
HsCIPK14	57.3	53.0	49.8	47.4	**45.7**	56.9	42.4	48.3	44.7
HsCIPK15	57.4	52.9	50.0	47.6	46.2	**56.7**	42.4	48.9	44.9
HsCIPK17	37.9	41.4	46.5	40.3	37.7	43.9	**57.5**	44.4	44.1
HsCIPK23	52.2	52.1	67.0	45.3	43.9	53.5	49.9	**77.1**	57.6
HsCIPK24	44.9	46.1	54.0	42.2	43.7	48.9	46.9	56.2	**68.9**
HsCIPK28	58.5	50.7	47.5	46.4	45.5	55.9	39.0	46.7	47.1
HsCIPK29	37.3	36.6	34.3	42.9	43.6	40.3	35.0	36.9	37.9
HsCIPK30	50.0	47.3	48.3	42.1	42.7	52.1	40.3	47.2	45.4
HsCIPK31	49.4	49.0	63.0	44.1	41.5	49.6	48.1	64.6	54.0

Figure S3. GenBank accession numbers and homology analysis of HsCIPKs.

a GenBank accession numbers for 12 *HsCIPK* genes.

b Homology comparison between HsCIPKs versus OsCIPKs.

c Homology comparison between HsCIPKs versus AtCIPKs.

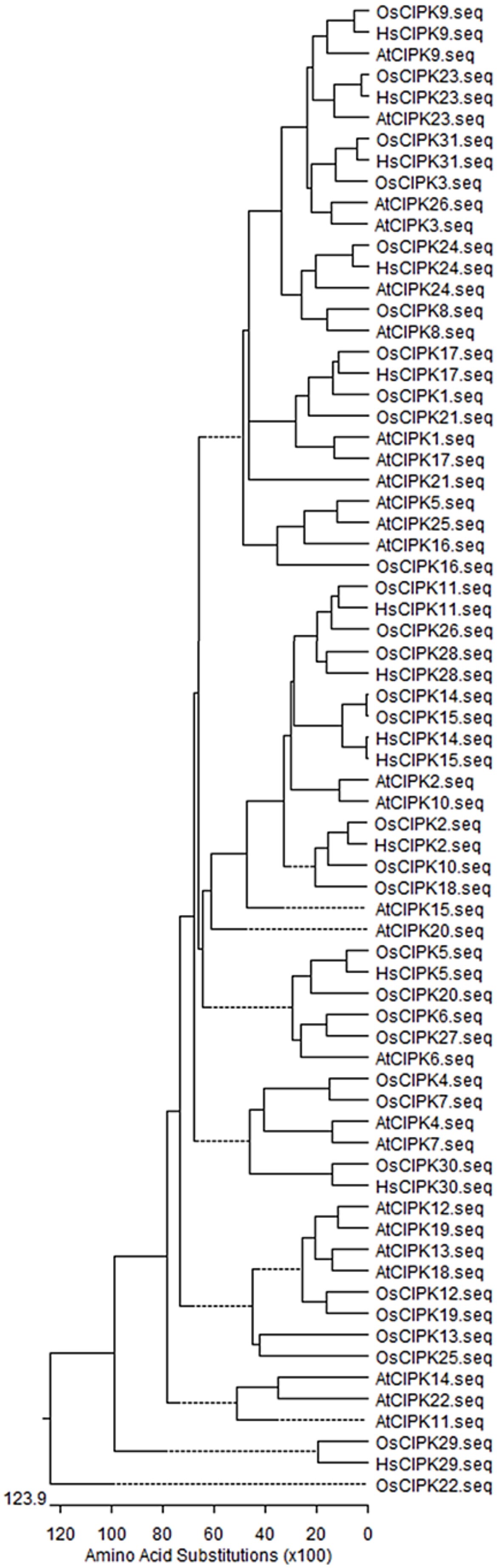

Figure S4. Phylogenetic analysis of HsCIPKs, OsCIPKs, and AtCIPKs.

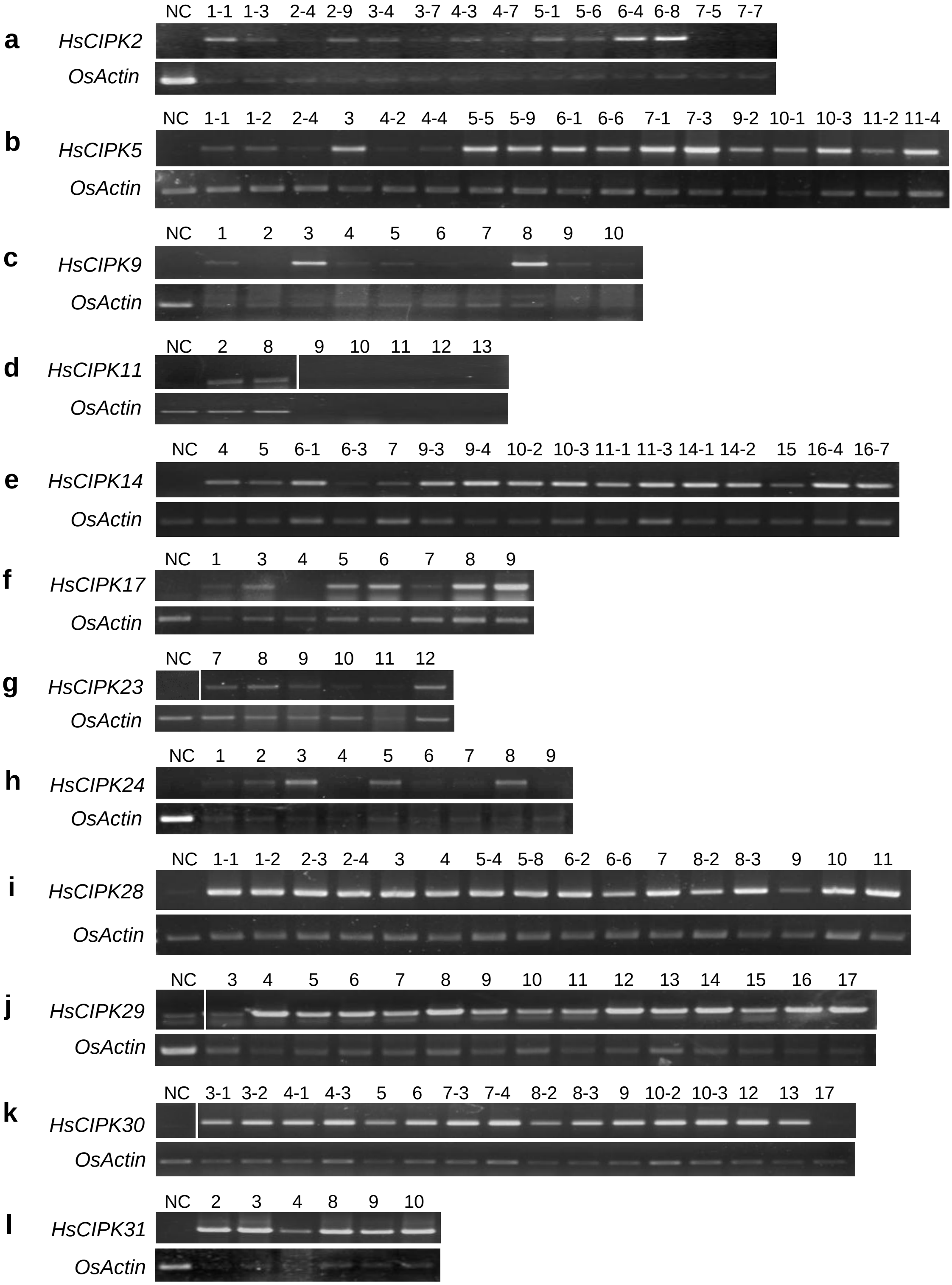

Figure S5. Molecular identification of *HsCIPK* overexpression in rice. RT-PCR assays were performed in T1 G418-resistant individual transgenic lines (**a-l**) under the control of 35S promoter. NC, the negative control (wild-type Nipponbare). Numbers show different transgenic lines whereas number-dash-numbers indicate the transgenic plants regenerated from the same callus.

Table S1. Accession numbers of rice and *Arabidopsis CIPK* cDNA in NCBI database.

Arabidopsis thaliana		*Oryza sativa*	
Gene	Accession No.	Gene	Accession No.
AtCIPK1	AF302112	*OsCIPK1*	EU703792
AtCIPK2	AF286050	*OsCIPK2*	EU703793
AtCIPK3	AF286051	*OsCIPK3*	Os07g48760
AtCIPK4	AY007221	*OsCIPK4*	NM_001073746
AtCIPK5	AF285105	*OsCIPK5*	EU703795
AtCIPK6	AF285106	*OsCIPK6*	AP004703
AtCIPK7	AF290192	*OsCIPK7*	AK111510
AtCIPK8	AF290193	*OsCIPK8*	NM_001049794
AtCIPK9	AF295664	*OsCIPK9*	AC104427
AtCIPK10	AF295665	*OsCIPK10*	EU703796
AtCIPK11	AF295666	*OsCIPK11*	EU703797
AtCIPK12	AF295667	*OsCIPK12*	EU703798
AtCIPK13	AF295668	*OsCIPK13*	AP003052
AtCIPK14	AF295669	*OsCIPK14*	AB264036
AtCIPK15	AF302111	*OsCIPK15*	AK121773
AtCIPK16	AY030304	*OsCIPK16*	AK061220
AtCIPK17	AY036958	*OsCIPK17*	EU703799
AtCIPK18	AY034099	*OsCIPK18*	EU703800
AtCIPK19	AY030303	*OsCIPK19*	AK069486
AtCIPK20	AY035225	*OsCIPK20*	AK107068
AtCIPK21	AY034100	*OsCIPK21*	NM_001066932
AtCIPK22	AF450478	*OsCIPK22*	AC117264
AtCIPK23	AY035226	*OsCIPK23*	EU703802
AtCIPK24	AF395081	*OsCIPK24*	AK102270
AtCIPK25	AF448226	*OsCIPK25*	AK065374
AtCIPK26	NM_180530	*OsCIPK26*	EU703805
		OsCIPK27	NM_001069722
		OsCIPK28	AC144743
		OsCIPK29	AK111746
		OsCIPK30	AK069231
		OsCIPK31	NM_001056484

Table S2. Primer sequences for *HsCIPKs* cloning.

Primer Name	Primer Sequences
HsCIPK2-SacI-F	TCCGAGCTCATGGGGGAGCAGAAGGGGAATATTCTGAT
HsCIPK2-XbaI-R	ACTTCTAGATCAACAAGATTGCTGCTGCGGCTGCGGC
HsCIPK5-KpnI-F	CGGGGTACCATGGAGAGGAAGTCCACCATCCTCATG
HsCIPK5-XbaI-R	TGCTCTAGATTAAATGACATTCCTTTTGGTCGACTT
HsCIPK9-SmaI-F	CCTCCCGGGATGGCTAGCAAATCGAAGATATACATGG
HsCIPK9-XbaI-R	TGCTCTAGACTAAAGCACATTCAGGATGCCTGTTAG
HsCIPK11-KpnI-F	CGGGGTACCATGGATGAGAGGAGGACTATATTGATGG
HsCIPK11-XbaI-R	TGCTCTAGATCATCCGATATTGCTAGCATGTGAATCTT
HsCIPK14-KpnI-F	CGGGGTACCATGGAGAACAGTGGGAAGATTGTAATG
HsCIPK14-XbaI-R	TGCTCTAGACTAACCATCAGCCCCATGCCAGGCCCAT
HsCIPK17-KpnI-F	ACCGGTACCAGCAGCGGGCAGCAGAAAGATG
HsCIPK17-XbaI-R	CCATCTAGATCACAAGGCAACCAGTGGTGTC
HsCIPK23-SmaI-F	CCTCCCGGGATGGTTGATTCGAGCGCGGGTGGGAAGA
HsCIPK23-XbaI-R	TGCTCTAGATCATGGAGACCTCCGGTGCCGGGTCT
HsCIPK24-SacI-F	ACCGAGCTCATGGCAGGCGCGCCGAGGAAGAAGCTGG
HsCIPK24-XbaI-R	TGCTCTAGACTAGCAAGTGGTTGTCCTCAGCAATGC
HsCIPK28-KpnI-F	CGGGGTACCATGGAAGACAGGAGTGTTTTGACCAAAC
HsCIPK28-XbaI-R	TGCTCTAGATTATTGTGGCAGTGGGGAGAAAGCATTTG
HsCIPK29-BamHI-F	AATGGATCCATGCCGTCCGCCTCCAGCGC
HsCIPK29-XbaI-R	ATTTCTAGATCACACTGCCTGAGGCTTCACGGCC
HsCIPK30-KpnI-F	CGGGGTACCATGGCCATGGAGAAGAACCAAGATTCC
HsCIPK30-XbaI-R	TGCTCTAGATCAGGAATTCGTGTTTGGCCTAGGCAC
HsCIPK31-SmaI-F	CCTCCCGGGATGTATCGGGCCAAGAGAGCCGCATTGT
HsCIPK31-XbaI-R	TGCTCTAGATCATGCCGCGGTGCTGTTATCTTCTAC

Table S3. Primer sequences for qRT-PCR assay in Tibetan Plateau annual wild barley.

Primer Name	Primer Sequences
qHsCIPK2-F	CACCGTGATTTGAAGCCCGAGAAC
qHsCIPK2-R	GATACCCAGCCATAAGCACAAAGAGGA
qHsCIPK5-F	GAAGGCAGCAGGGCAGAACAACCA
qHsCIPK5-R	TGGATCCCTGCAGCGTCACAAGTC
qHsCIPK9-F	TTATTTCAAGGTCGGCGGGATTCAA
qHsCIPK9-R	ACCTTTTCTTCCTGTCTTGTCACCTTTCA
qHsCIPK11-F	ACCGAGCCTGAAGCAACGCACAC
qHsCIPK11-R	AAACCGTTCTTCCCTGCGACCAT
qHsCIPK14-F	GCCCGAGAACCTATTGCTTGATGA
qHsCIPK14-R	AACAGGATGACACCGCAAGACCAGA
qHsCIPK17-F	GTGAGCTATTGCCATGGAAAGGGTGTCTAC
qHsCIPK17-R	TACTCCACAAGACCAGATATCCGACAATGA
qHsCIPK23-F	ATTTTGGACTCAGCGCCCTATCG
qHsCIPK23-R	ATCGGCCTTGGCTCCATCATAACC
qHsCIPK24-F	GCAGCAGATATTTGGTCATGTGGTGTT
qHsCIPK24-R	GATCATTGACTTGGCCCCTGGAGAG
qHsCIPK28-F	GCGGCGCCGAGGGAAGGAAAGA
qHsCIPK28-R	CTGCTGAACATCGCCGTGCCATCC
qHsCIPK29-R	AACCCGGCCACGCGCATTGACA
qHsCIPK29-F	GTCCCGCGCCATGTCCTCCTTGAA
qHsCIPK30-F	GCCCGTCGCTCGCCGCTACTTTC
qHsCIPK30-R	CGTGCGCGTCGAGGAGGAGGTTCT
qHsCIPK31-F	CACCTCGCAATGCCCTCCAAAAG
qHsCIPK31-R	CTTCGCCTTTTTCAGCTCAACCACA
qHvActin-F	CCAAAAGCCAACAGAGAGAA
qHvActin-R	GCTGACACCATCACCAGAG

Table S4. Primer sequences for RT-PCR assay in rice transgenic lines.

Primer name	Primer sequences
HsCIPK2-F	TGACGGCGCCAAGGCTGACATCT
HsCIPK2-R	TTAACATGGTTGCTGCTGCGGCTG
HsCIPK5-F	ATGGAGAGGAAGTCCACCATCCTCATG
HsCIPK5-R	TTAAATGACATTCCTTTTGGTCGACTT
HsCIPK9-F	ATGGCTAGCAAATCGAAGATATACATGG
HsCIPK9-R	CTAAAGCACATTCAGGATGCCTGTTAG
HsCIPK11-F	ATGGATGAGAGGAGGACTATATTGATGG
HsCIPK11-R	TGGTGGTTGTTCCTGCTGCTCCTG
HsCIPK14-F	ATGGAGAACAGTGGGAAGATTGTAATG
HsCIPK14-R	CGGTACATCTCTATCAAGTTCTGACCTT
HsCIPK17-F	ATGGTGGCGACGGGCGACGC
HsCIPK17-R	TACTCCACAAGACCAGATATCCGACAATGA
HsCIPK23-F	ATGGTTGATTCGAGCGCGGGTGGGAAGA
HsCIPK23-R	TCATGGAGACCTCCGGTGCCGGGTCT
HsCIPK24-F	ATGGCAGGCGCGCCGAGGAAGAAGCTGG
HsCIPK24-R	CTAGCAAGTGGTTGTCCTCAGCAATGC
HsCIPK28-F	AAGTGGCGAGATGGTCTGCTCCACACCG
HsCIPK28-R	ACCGGGCCTCCCTTTGACTATATCTTTC
HsCIPK29-F	ATGCCGTCCGCCTCCAGCGC
HsCIPK29-R	TCACACTGCCTGAGGCTTCACGGCC
HsCIPK30-F	ATGGCCATGGAGAAGAACCAAGATTCCAAAG
HsCIPK30-R	ATGGTGATCCGGGTGTTGGGGTTCG
HsCIPK31-F	GCACCATCGGGGAAGGGACATT
HsCIPK31-R	CTTCGCCTTTTTCAGCTCAACCACA
*OsActin*2-F	GGAACTGGTATGGTCAAGGC
*OsActin*2-R	AGTCTCATGGATACCCGCAG

Table S5. Summary of endogenous *HsCIPK* responses to heavy metal toxicities.

Gene	20 μM $HgCl_2$ (roots)	20 μM $CdCl_2$ (roots)	0.5 mM $K_2Cr_2O_7$ (roots)	20 μM $PbCl_2$ (roots)	1 mM $CuSO_4$ (roots)
HsCIPK2	↑ (6.6±0.19) ***	↑ (7.6±0.25) ***	↑ (3.9±0.10) ***	↑ (1.8±0.03) ***	↑ (3.0±0.20) ***
HsCIPK5	↑ (2.2±0.36)	↑ (2.3±0.03) ***	↑ (7.9±0.54) ***	↑ (1.8±0.32)	↑ (14.1±1.22) ***
HsCIPK9	—	↑ (11.5±0.57) ***	↑ (4.5±0.54) **	↑ (6.1±0.71) **	↑ (2.91±0.14) ***
HsCIPK11	↑ (3.6±0.33) **	↑ (4.0±0.05) ***	↑ (4.8±0.19) ***	↑ (1.48±0.53) **	↑ (4.3±0.59) **
HsCIPK14	↑ (4.7±0.48) **	↑ (13.3±1.24) ***	↑ (4.2±0.10) ***	↑ (4.8±0.53) **	↓ (0.59±0.03) ***
HsCIPK17	↑ (30.6±2.40) ***	↑ (70.9±1.76) ***	↑ (15.7±0.62) ***	↑(6.7±0.68) **	↑ (12.7±0.77) ***
HsCIPK23	↑ (10.7±0.49) ***	↑ (5.2±0.16) ***	↑ (4.8±0.25) ***	↑ (2.6±0.18) ***	↑ (3.1±0.09) ***
HsCIPK24	↑ (6.4±0.59) ***	↑ (7.1±0.39) ***	↑ (8.1±0.32) ***	↑ (3.3±0.36) **	↑ (1.8±0.12) **
HsCIPK28	↑ (4.0±1.24) *	—	↓ (0.55±0.07) **	↑ (2.3±0.16) **	↑ (2.6±0.61) *
HsCIPK29	↑ (9.5±0.78) ***	↑ (66.6±2.6) ***	↑ (6.1±0.46) ***	↑ (4.2±0.03) ***	↑ (6.9±0.24) ***
HsCIPK30	↑ (12.9±1.41) **	↑ (3.9±0.46) **	↑ (12.1±1.25) **	↑ (1.9±0.15) **	↑ (14.3±3.11) **
HsCIPK31	↑ (5.5±0.06) ***	↑ (69.5±8.32) **	↓ (0.4±0.03) ***	↓ (0.46±0.01) ***	↑ (7.1±0.50) ***

↑, ↓, and − indicate elevation, reduction, and no changes at *HsCIPK* mRNA levels relative to the 0-h time point, respectively. The expression levels of *HsCIPKs* being three times higher than the mock control are shown in Fig. 2 and Fig. 3.The numbers in brackets show the maximum levels of elevation or reduction at the *HsCIPK* mRNA levels relative to the corresponding mock control (0-h time point) during treatments.Single, double, and triple asterisks indicate $P < 0.01$, 0.001, and 0.0001, respectively (t test; compared to the 0-h time point mock control).

Table S6. Summary of endogenous *HsCIPK* responses to other abiotic stresses.

Gene	400 mM NaCl (roots)	20% PEG 6000 (seedlings)	20 µM $AlCl_3$ (roots)	4°C Cold (seedlings)	35°C Heat (seedlings)	20 µM ABA (roots)
HsCIPK2	↑ (4.0±0.25) ***	↑ (1.3±0.05)	↑ (2.0±0.01) ***	↓ (0.83±0.18)	↓ (0.38±0.05) *	↑ (3.8±0.46) **
HsCIPK5	↑ (2.9±1.10) *	↑ (1.29±0.22)	—	—	↑ (2.9±0.94) *	↑ (1.8±0.46)
HsCIPK9	—	↑ (3.0±0.02) ***	↓ (0.27±0.01) ***	↑ (10.6±1.0) ***	↑ (2.1±0.06) ***	↑ (1.7±0.67) *
HsCIPK11	↑ (1.5±0.12) *	↑ (1.5±0.01) **	↑ (1.7±0.03) ***	—	↓ (0.7±0.05) **	↑ (1.5±0.01) **
HsCIPK14	↓ (0.39±0.01) ***	↓ (0.2±0.01) ***	—	—	↓ (0.4±0.01) ***	↓ (0.78±0.08) *
HsCIPK17	↑ (3.2±0.11) ***	↓ (0.8±0.02) **	↑ (1.6±0.14) *	—	↑ (1.5±0.15) *	↑ (7.4±0.42) ***
HsCIPK23	—	↓ (0.8±0.06) *	↓ (0.6±0.03) ***	—	↓ (0.87±0.07)	—
HsCIPK24	↑ (3.1±0.38) **	↑ (1.2±0.03) **	↑ (2.3±0.21) **	↓ (0.89±0.11)	↑ (1.3±0.10) *	↓ (0.77±0.06) *
HsCIPK28	↓ (0.76±0.16) *	↑ (1.9±0.3) *	↑ (2.9±0.09) *	—	↑ (1.4±0.45)	↓ (0.90±0.11)
HsCIPK29	↑ (3.2±0.06) **	↑ (2.5±0.31) *	↑ (2.7±0.45) *	—	↑ (1.4±0.34)	—
HsCIPK30	↑ (10.1±0.40) ***	—	↑ (4.3±0.23) ***	↑ (2.8±0.19) ***	—	—
HsCIPK31	↑ (2.6±0.05) ***	↑ (3.7±0.02) ***	↑ (4.8±0.06) ***	↑ (5.8±0.30) ***	↓ (0.8±0.07) *	↑ (5.4±0.72) **

↑, ↓, and − indicate elevation, reduction, and no changes at *HsCIPK* mRNA levels relative to the 0-h time point, respectively. The expression levels of *HsCIPKs* being two times higher than the mock control are shown in Fig. 4. The numbers in brackets show the maximum levels of elevation or reduction at the *HsCIPK* mRNA levels relative to the corresponding mock control (0-h time point) during treatments. Single, double, and triple asterisks indicate $P < 0.01$, 0.001, and 0.0001, respectively (t test; compared to the 0-h time point mock control).

Table S7. Summary of root growth tolerance to abiotic stresses in rice transgenic lines overexpressing *HsCIPKs.*

Gene	0.5 µM $HgCl_2$	5 µM $CdCl_2$	5 µM $K_2Cr_2O_7$	5 µM $PbCl_2$	0.25 µM $CuSO_4$	50 mM NaCl	10% PEG 6000	1 µM ABA	50 µM $AlCl_3$
HsCIPK2	↑*	—	↑*	—	—	↑*	—	↑*	—
HsCIPK5	—	—	↑ *	—	↑**	↑**	—	—	—
HsCIPK9	—	↑*	↑*	—	—	—	—	—	↓**
HsCIPK11	—	↑**	—	—	—	—	—	—	—
HsCIPK14	↑*	↑**	—	—	—	—	—	—	↓*
HsCIPK17	↑*	↑**	↑*	↓*	—	↑*	↑*	↑**	↓*
HsCIPK23	—	↑*	—	—	—	—	↑*	—	—
HsCIPK24	—	↑***	—	—	↑*	—	—	↓*	—
HsCIPK28	—	—	—	↓*	—	↑*	—	—	—
HsCIPK29	—	↑*	—	↓*	↑*	↑**	↑**	↓*	—
HsCIPK30	—	—	↑*	↓*	—	↑**	—	—	↓*
HsCIPK31	—	—	↓*	—	↑*	—	↑**	—	—

↑, ↓, and − indicate elevation, reduction, and no changes in root growth tolerance to stress treatments relative to the wild-type control NT seedlings, respectively. The detailed data are shown in Fig. 5 and Fig. 6. The numbers in brackets show the maximum levels of elevation or reduction at the *HsCIPK* mRNA levels relative to the corresponding mock control (0-h time point) during treatments. Single, double, and triple asterisks indicate $P < 0.05$, 0.01, and 0.001, respectively (*t* test; compared to the wild-type control NT seedlings).
